# Is Transvaginal Minimally Invasive Sacrospinous Ligament Fixation a Safe and Effective Surgical Approach for Treating Recurrent Apical Pelvic Organ Prolapse?

**DOI:** 10.3390/jcm14155235

**Published:** 2025-07-24

**Authors:** Jonatan Neuman, Asnat Groutz, Menahem Neuman, Ronen S. Gold

**Affiliations:** 1 Medical School, Semmelweis University, 1085 Budapest, Hungary; 2 Urogynecology Unit, Lis Women Hospital, Tel Aviv Medical Center, Gray Faculty of Medical and Health Sciences, Tel Aviv University, Tel Aviv 6423906, Israel; agroutz@yahoo.com; 3 The Urogynecology Service, Assuta Medical Centers, Ben Gurion University Medical School, Be’er Sheva 8410501, Israel; menahem.neuman@gmail.com

**Keywords:** apical pelvic organ prolapse, sacrospinous ligament fixation, recurrent pelvic organ prolapse, outcomes results, lower urinary tract symptoms, quality of life, enplace device

## Abstract

**Background:** Recurrent apical pelvic organ prolapse (POP) presents significant management challenges, with limited evidence on optimal surgical approaches. This study evaluated the safety and long-term effectiveness of minimally invasive sacrospinous ligament (SSL) fixation using the EnPlace^®^ device for treating recurrent apical POP. **Methods:** A cohort analysis was performed on 82 consecutive patients (mean age 65.9 ± 8.6 years) with stage III or IV recurrent symptomatic apical POP. All patients underwent transvaginal SSL fixation using the EnPlace^®^ device between January 2021 and July 2023. Primary outcomes included anatomical cure rates, patient satisfaction, and complications. Long-term follow-up was conducted via a structured telephone survey in December 2024. **Results:** The mean interval between primary and recurrent repair was 3.2 ± 2.6 years. Most patients (64.6%) underwent surgery under regional anesthesia with a mean operative time of 24.1 ± 7.1 min and minimal blood loss (23.8 ± 6.5 mL). No intraoperative complications occurred, and 98.8% of patients were discharged the same day. Two early postoperative complications occurred, neither requiring surgical intervention. At six-month follow-up, significant improvements were observed in POP-Q measurements for apical prolapse, cystocele, and rectocele. Long-term follow-up (mean 31.6 ± 8.3 months) revealed that only 11 patients (13.4%) reported mild POP symptoms. Patient satisfaction scores averaged 90.8 ± 17.1, with only 8.5% reporting low satisfaction. Only two patients (2.4%) required additional intervention for recurrent apical POP. **Conclusions:** Minimally invasive SSL fixation using the EnPlace^®^ device demonstrates favorable safety and efficacy for recurrent apical POP, offering a viable alternative to more invasive procedures with high patient satisfaction and low recurrence rates.

## 1. Introduction

Pelvic organ prolapse (POP) is a prevalent condition, affecting approximately 50% of women who have given birth. The causes of POP are complex, involving mechanical and genetic factors, as well as age-related degeneration. Major obstetric risk factors include vaginal childbirth, especially traumatic deliveries such as prolonged labor, high birth weights, instrumental-assisted deliveries, and advanced maternal age. Additional contributing factors encompass genetic connective tissue disorders, chronic conditions associated with increased intra-abdominal pressure (such as chronic obstructive pulmonary disease, chronic constipation, and obesity), previous pelvic surgery, and hormonal changes associated with menopause [1,2,3,4,5]. The anatomical support of the pelvic organs relies on a sophisticated three-level support system, as described by DeLancey [6,7]. Level I support involves the cardinal and uterosacral ligament complex, providing apical suspension. Level II support includes the pubo-cervical and recto-vaginal fascia, along with its lateral attachments to the pelvic sidewall via the arcus tendineus fasciae pelvis. Level III support consists of the perineal membrane and external anal sphincter, providing distal support. Compromise of any of these levels can result in specific types of prolapse, with Level I defects leading to apical POP. The impact of POP on quality of life is profound, affecting physical, psychological, social, and sexual domains of a woman’s life. Research has consistently demonstrated that women with POP experience significantly reduced quality of life scores compared to age-matched controls without prolapse, with symptom severity directly correlating with quality of life deterioration as measured by validated instruments such as the Pelvic Floor Distress Inventory (PFDI) and Pelvic Floor Impact Questionnaire (PFIQ) [8,9,10]. The unpredictable nature of symptoms often leads to activity restriction and social isolation, as women may avoid situations where they cannot readily access bathroom facilities or where physical exertion might exacerbate their condition [11,12]. The chronic nature of the POP and uncertainty about treatment outcomes contribute to ongoing psychological distress [13,14]. The severity of quality-of-life impairment often correlates with the stage of prolapse, with more advanced stages causing greater functional limitations and distress. This relationship underscores the importance of early intervention and comprehensive management approaches that address not only the anatomical defect but also the broader impact on women’s lives [15,16]. Given these multidimensional impacts, quality of life improvement has become one of the primary outcomes in the management of POP, with surgical interventions showing potential to improve women’s overall well-being and functional capacity significantly.

Apical POP occurs as a result of a level I support defect. The clinical presentation of apical POP varies considerably among patients but commonly includes a sensation of pelvic pressure or heaviness, a visible or palpable vaginal bulge, lower urinary tract symptoms (LUTS), and sexual dysfunction. Many patients report that symptoms worsen with prolonged standing, physical exertion, or straining, and improve with rest or lying down. These symptoms significantly limit a woman’s ability to participate in exercise, recreational activities, and occupational tasks, thereby reducing work productivity and daily functioning. Additionally, many women experience embarrassment, anxiety, and depression related to their condition, often reporting feeling “abnormal” or “damaged.” This leads to social withdrawal, reduced self-esteem, body image concerns, and reluctance to seek medical care. Apical prolapse, which can affect either the uterus or the vaginal vault in patients who have undergone a hysterectomy, poses significant management challenges due to its complexity and impact on pelvic function [17,18]. The apical compartment serves as the cornerstone of pelvic support, and its failure often leads to multicompartmental prolapse involving the anterior and posterior vaginal walls as well. This anatomical relationship underscores the importance of adequate apical support restoration in achieving durable surgical outcomes [19,20].

Transabdominal robotic or laparoscopic sacropexy (SCP), with or without uterine preservation, is considered an effective surgery with a long-term success rate of over 80% [21,22]. The transabdominal approach requires general anesthesia, extends surgery time, and necessitates specialized instruments, resulting in higher surgical costs. Additionally, this technique may lead to complications related to mesh implantation. Transvaginal repair is an alternative surgical approach to correct apical POP. The employment of transvaginal synthetic mesh in pelvic floor repair has been restricted due to regulatory limitations governing the use of synthetic materials in these procedures [23]. Transvaginal sacrospinous ligament (SSL) fixation is a well-established technique for addressing apical POP. This procedure is effective in restoring apical support and can be performed either unilaterally or bilaterally. However, it necessitates considerable dissection to access the SSL and demands a high level of surgical skills. The EnPlace^®^ device presents a minimally invasive alternative to the traditional SSL access. Clinical studies have demonstrated its safety and efficacy, allowing for SSL fixation without the complexities of extensive pelvic dissection, general anesthesia, or the use of synthetic mesh implants [24,25,26].

Primary apical POP repair is associated with recurrence rates ranging from 10% to 30% [27,28]. The observed elevated recurrence rates can be primarily attributed to inadequate surgical techniques that compromise the fixation of critical supportive structures. Furthermore, the natural degeneration of connective tissue and ligaments associated with aging contributes significantly to these outcomes. Additional factors, such as the presence of high-risk medical comorbidities, including obesity, chronic constipation, smoking, and connective tissue disorders, may further exacerbate the situation [29,30,31]. The surgical management of recurrent apical POP continues to provoke discussion among specialists, particularly regarding the utilization of mesh versus autologous tissue for repair. Recurrent repairs must contend with a variety of complications, including altered pelvic anatomy, compromised tissue integrity, the presence of scar tissue, and potential issues such as mesh contraction or scarring [32]. These factors can result in anatomical distortion, complicating the surgical approach and potentially impacting patient outcomes. Furthermore, the increased likelihood of recurrence associated with secondary repairs as opposed to primary interventions remains a key concern in optimizing patient outcomes.

The present study investigated the safety and long-term effectiveness of minimally invasive SSL fixation utilizing the Enplace^®^ device in patients with recurrent apical POP.

## 2. Methods

A cohort analysis was performed on 82 patients diagnosed with recurrent symptomatic apical POP following previous reconstructive surgery. The severity of the prolapse was assessed using the Pelvic Organ Prolapse Quantification (POP-Q) system [33]. A recurrent apical POP was categorized as significant when classified as stage III or IV based on the POP-Q criteria. All participants were subjected to transvaginal surgical repair employing the Enplace^®^ device for SSL fixation. Exclusion criteria for using the Enplace^®^ device included active malignancy, ongoing evaluation of uterine or cervical pathology, reproductive tract anomalies, previous pelvic radiation therapy, active pelvic inflammatory disease, or a known allergy to nickel or nitinol. All surgeries were performed by experienced urogynecologists from January 2021 to July 2023. The Institutional Review Board (IRB) approved the study protocol (ASMC 0039-24). The IRB waived the requirement for obtaining a formal written informed consent from the patients because the study involved a retrospective data analysis and a follow-up telephone questionnaire.

The EnPlace^®^ device (FEMSelect, Tel Aviv, Israel) is an innovative system that was developed to address the surgical limitations of traditional SSL fixation techniques, which require extensive dissection, carry risks of neurovascular injury, and necessitate considerable surgical expertise. The EnPlace^®^ system consists of several key components designed to work synergistically: Firstly, a Nitinol anchor manufactured from biocompatible Nickel–Titanium alloy. This material is selected for its shape-memory properties and excellent tissue integration characteristics. Nitinol is widely used across multiple disciplines as vascular clips, stents, and orthopedic anchors. The material is safe for MRI imaging [34]. The anchor features a helical configuration that provides secure fixation within the SSL while minimizing tissue trauma during insertion. Secondly, a delivery system with a precise guidance mechanism that accurately targets the SSL through a minimally invasive transvaginal approach. These key features facilitate surgical depth control, ensuring accurate anchor placement while preventing over-penetration, thereby minimizing the risk of neurovascular injury. The procedure can be performed using either regional or general anesthesia. Regional anesthesia has the advantage of reducing anesthesia-related morbidity when appropriate and facilitating a faster recovery [35]. Patients are positioned in the dorsal lithotomy position with appropriate padding to ensure optimal visualization and access. The surgical approach begins with careful identification of anatomical landmarks and assessment of the type and severity of POP. The device is positioned using integrated guidance, and the anchor is deployed directly into the SSL. The sutures are securely attached to the apical tissues, either the uterine cervix or the vaginal vault, using permanent suture material. Tension is then adjusted to achieve the appropriate anatomical restoration. Finally, the vaginal epithelium is closed in layers with absorbable sutures. A concomitant anterior or posterior native-tissue repair was undertaken for patients presenting with cystocele and/or rectocele, respectively. Those diagnosed with stress urinary incontinence (SUI) underwent a concomitant inside-out transobturator mid-urethral sling (MUS) procedure (Serasis^®^, Serag-Wiessner, Naila, Germany) [36].

Demographic, clinical, intraoperative, and early postoperative data were retrospectively retrieved from a computerized database. Preoperative data included age, parity, BMI, smoking status, severe comorbidities, LUTS, and severity of POP. Intraoperative data included the duration of surgery (minutes), estimated blood loss (mL), and surgical complications. A follow-up assessment was carried out at six weeks and six months postoperatively. The postoperative evaluation included anatomical and functional cure rates, pain, dyspareunia, LUTS, and urinary tract infections (UTIs). Anatomical cure was defined as no apical prolapse beyond stage II by the POP-Q classification and no significant POP-related symptoms.

During December 2024, a long-term follow-up was conducted through a structured telephone survey. Patient-reported outcomes were assessed based on decision regret or satisfaction using the Satisfaction with Decision Scale for Pelvic Floor Disorders, rated from 0 to 100 [37]. A low satisfaction score was defined as a satisfaction score of 60 or lower. Severity of subjective POP symptoms was assessed by a 0 to 10 analogue scale with 0 for no symptoms and 10 for severe symptoms.

Statistical analysis was conducted using the Student’s *t*-test for continuous data and Fisher’s exact test for categorical data. The results are summarized as mean ± standard deviation (SD) or percentages, depending on the variable type. All statistical tests were two-sided, with a *p*-value of less than 0.05 considered statistically significant. SPSS software version 27 (IBM Corporation, Armonk, NY, USA) was used for statistical analysis.

## 3. Results

A cohort of 82 consecutive patients (mean age 65.9 ± 8.6 years; range 41–78 years) with stage III or IV recurrent apical POP was investigated. The primary apical repair consisted of vaginal hysterectomy with concomitant uterosacral fixation in 57 (69.5%) patients, and uterine-preserving procedures, including transvaginal mesh, SSL fixation, and the Manchester operation, in 25 (30.5%). All patients underwent recurrent surgical repair using transvaginal SSL fixation with the EnPlace^®^ device. Additionally, 69 patients (84.1%) underwent concomitant native tissue colporrhaphies, while 13 patients (15.9%) also had a MUS procedure performed. The mean interval between the initial apical pelvic prolapse surgery and the subsequent surgical repair was 3.2 ± 2.6 years, with a reported range spanning from 0.1 to 10 years.

The demographic and clinical characteristics of the study cohort are presented in Table 1. Preoperatively, all participants reported significant bulging symptoms. Furthermore, 16 patients (19.5%) presented with concomitant SUI, 22 (26.5%) exhibited overactive bladder (OAB) symptoms, and two patients (2.4%) experienced defecatory dysfunction. The clinical characteristics of patients who had previously undergone vaginal hysterectomy were similar to those of patients who underwent uterine-preserving procedures. The only exception was the interval between the primary surgery and the subsequent repair of recurrent apical POP, which was 3.5 ± 3.1 years for the hysterectomy group and 2.1 ± 1.6 years for the uterine-preserving group (*p* = 0.039).

There were no intraoperative complications reported. Most (64.6%) patients underwent the transvaginal SSL fixation under regional anesthesia, while the remaining patients received general anesthesia. The mean operative time was 24.1 ± 7.1 min, with an average intraoperative blood loss of 23.8 ± 6.5 mL recorded. All patients, except for one, were discharged on the same day as their surgery.

There were two cases of early postoperative complications, none of which required surgical intervention. A 74-year-old patient experienced paroxysmal atrial fibrillation (PAF) immediately postoperatively, for which she was initiated on full anticoagulation therapy. Several hours later, she developed a retroperitoneal hematoma, which resulted in a significant drop in hemoglobin from 12 g/dL to 7 g/dL. A CT angiography was performed, revealing no evidence of active bleeding. The patient was managed conservatively with blood transfusions and did not require any further surgical intervention. A second patient, who underwent concomitant colporrhaphies and a MUS procedure, experienced postoperative urinary retention. This condition was managed conservatively through transurethral catheterization over a period of one week, resulting in complete resolution of the urinary retention following the intervention.

The preoperative, six-week, and six-month postoperative POP-Q measurements are summarized in Table 2. The objective POP-Q data show a statistically significant improvement in point C (apical prolapse), point Ba (cystocele), and point Bp (rectocele) at the six-month follow-up, regardless of the method used for the previous primary apical POP repair.

The long-term follow-up outcomes are summarized in Table 3. The average follow-up duration was 31.6 ± 8.3 months (range 17–59 months). Out of the 82 patients, only 11 (13.4%) reported experiencing mild symptoms of POP, with a mean severity score of 3.9 on a scale from 0 to 10 (ranging from 2 to 7). The clinical characteristics, demographics, and intraoperative course of patients with recurrent POP symptoms were similar to those without such symptoms. Patient-reported outcomes were evaluated using a global satisfaction scale ranging from 0 to 100, resulting in a mean satisfaction score of 90.8 ± 17.1. Only seven patients (8.5%) reported low satisfaction scores of less than 60. None of the patients developed new-onset OAB symptoms, dyspareunia, or defecation disorders. Only two patients (2.4%) required additional intervention for clinically significant recurrent apical POP. The first patient was a 54-year-old woman who developed apical POP after undergoing a laparoscopic hysterectomy for a fibroid uterus. She received Enplace^®^ SSL fixation, which had an unremarkable short-term follow-up. However, approximately two years postoperatively, she presented with both a cystocele and vault prolapse, leading to an anterior colporrhaphy augmented by transvaginal mesh implantation for enhanced apical support. The second patient was an 81-year-old woman who underwent Enplace^®^ SSL fixation following a vaginal hysterectomy. One year postoperatively, she experienced recurrent vault prolapse and chose conservative management with a vaginal pessary.

## 4. Discussion

Pelvic floor disorders represent a significant health burden affecting millions of women worldwide, with prevalence increasing substantially with age. The global impact of these common disorders is associated with substantial healthcare costs and resource utilization. Epidemiological studies suggest that the lifetime risk of undergoing surgery for pelvic floor disorders, either POP or SUI, approaches 20% with a significant proportion of patients requiring repeat interventions due to recurrence of POP, or surgical-associated complications [38]. Apical POP, with either uterine prolapse or vaginal vault prolapse, constitutes a particularly challenging subset that requires specialized surgical expertise and careful consideration of multiple treatment options. The management of apical POP is complex due to the apex’s essential role in supporting the entire vaginal axis and affecting the function of nearby compartments. When apical support is not adequately addressed, it often results in the recurrence of not only apical prolapse but also the secondary development of anterior and posterior compartment defects [39]. Recurrent apical POP remains one of the main reasons for reoperation in patients with POP, making effective correction of the vaginal apex essential for durable repair in these women.

The surgical management of apical POP includes various techniques, each with distinct advantages and disadvantages [17,18,19,20,21]. These techniques should be selected based on the patient’s unique anatomy, preferences, and risk of complications. Transabdominal approaches, including laparoscopic and robotic SCP, are currently considered the gold standard for apical POP repair, providing high success rates and durable results. These procedures necessitate advanced surgical skills, general anesthesia, and laparoscopic or robotic equipment, which contributes to their high costs and limits accessibility in some clinical settings. Additionally, while the use of either transabdominal or transvaginal synthetic mesh provides excellent structural support, it carries inherent risks including mesh exposure, erosion, and chronic pain. The two primary transvaginal natural tissue apical repairs are SSL fixation and uterosacral ligament (USL) suspension. Traditional SSL fixation requires significant pelvic dissection, which poses risks of vascular and neurovascular injury, particularly to the pudendal neurovascular bundle. The success of the procedure primarily relies on the surgeon’s experience and thorough understanding of pelvic anatomy [40]. Early complications of SSL fixation may include hematoma formation, infection, and urinary retention. Injuries to the pudendal nerve may result in chronic pain syndromes that necessitate a multimodal approach to pain management [41,42]. Additionally, sexual dysfunction may occur as a result of vaginal deviation if fixation is performed on only one side [43,44]. The USL suspension technique provides an alternative method for using native tissue, offering the benefit of maintaining the normal vaginal axis. However, this approach can be technically difficult, especially in cases where the ligaments are severely weakened or absent, a situation often seen in patients with recurrent prolapse [45,46]. The USL suspension carries a higher risk of ureteral injury compared to SSL fixation, particularly in patients undergoing concomitant anterior colporrhaphy. This technique is also less effective for patients with vaginal vault prolapse following a hysterectomy [47].

Despite advances in surgical techniques for POP repair, primary surgical failure remains a significant clinical problem, with reported POP recurrence rates varying from 10 to 30% depending on the procedure performed and duration of follow-up. Apical recurrence after primary apical POP repair follows predictable patterns influenced by the surgical technique used and specific patient factors: (1) technical factors, such as inadequate fixation strength, inappropriate suture placement, or insufficient tissue quality at the fixation site; (2) patient factors, such as advanced age, obesity, chronic cough, constipation, and connective tissue disorders; and (3) anatomical factors, such as loss of anterior or posterior compartment support leading to recurrent multicompartmental prolapse [27,28]. Additionally, the durability of SSL fixation appears to be influenced by the specific technique employed, with bilateral fixation showing superior long-term outcomes compared to unilateral approaches. The surgical management of recurrent apical POP presents unique anatomical, technical, and patient-centered challenges. It requires careful consideration of several factors, including the patient’s age, BMI, comorbidities, previous surgical techniques, and a comprehensive evaluation of the remaining anatomical support structures. However, the available data regarding outcomes are limited and inconsistent. There are several surgical options available for the correction of recurrent apical POP, with repeat transabdominal SCP (re-SCP) being the most commonly used. Re-SCP is associated with high rates of anatomical success and a lower risk of recurrence. However, it requires general anesthesia and advanced surgical skills due to the potential for scar tissue and dense adhesions from previous surgeries. Further, current literature mainly consists of small patient cohort studies focusing on re-SCP, resulting in inadequate data regarding optimal management strategies for recurrent apical POP. This gap in evidence significantly hinders the clinical decision-making process for both surgeons and their patients.

Bauters et al. [48] conducted a study comparing 39 women who underwent re-laparoscopic SCP with 156 women who had a primary laparoscopic SCP for symptomatic apical POP. The primary outcome measured was the rate of intraoperative and early postoperative complications occurring within the first three months. Secondary outcomes included subjective and objective success rates, surgical variables, mesh-related complications, and the need for reintervention. All participants had a minimum follow-up duration of 12 months. The study found no significant differences in the rates of intraoperative and early postoperative complications; however, the conversion rate to laparotomy was higher for re-SCP cases (10.3%) compared to primary SCP cases (0.6%). At long-term follow-up, there were no notable differences in mesh-related complications (re-SCP: 17.9% vs. primary SCP: 9.6%, *p* = 0.14), the need for reintervention due to complications (re-SCP: 12.8% vs. primary SCP: 5.1%, *p* = 0.14), or POP recurrence (re-SCP: 15.4% vs. primary SCP: 8.3%, *p* = 0.18). Subjective and objective success rates were also found to be comparable between the two groups. The authors concluded that re-SCP is as safe and effective as primary SCP; however, the risk of conversion to open laparotomy is higher in re-SCP cases. Studer et al. [49] studied 377 women who underwent a primary laparoscopic SCP. Ten women presented with a symptomatic recurrent prolapse requiring further surgical intervention. A re-SCP was performed in eight women, including two with additional laparoscopic paravaginal repair to correct the displaced mesh placement at initial surgery. The investigators concluded that individualized re-SCP after primary SCP is a challenging yet feasible and safe surgical option. Ruess et al. [50] published a series of seven patients who developed recurrent POP stage II or greater after SCP. They concluded that laparoscopic revision of SCP for recurrent apical POP is feasible but needs to be individualized, as the number of cases is limited, and available evidence is scarce.

The present study was conducted to evaluate the long-term surgical outcomes of transvaginal minimally invasive SSL fixation in a cohort of 82 patients (mean age 65.9 ± 8.6 years, range 41–78 years) with recurrent stage III or IV apical POP after previous reconstructive pelvic surgery. All patients underwent bilateral transvaginal SSL fixation utilizing the EnPlace^®^ device. The EnPlace^®^ device provides several important advantages over traditional SSL fixation techniques. This unique device allows for SSL fixation without extensive dissection, general anesthesia, or synthetic mesh implantation. The procedure usually takes only 20 to 30 min, compared to 60 to 90 min for conventional methods. This shorter duration leads to less exposure to anesthesia, lower operative costs, and faster patient recovery. The controlled placement of anchors minimizes variability between surgeons and contributes to more consistent outcomes. Additionally, the reduced surgical trauma and the option for regional anesthesia allow for same-day discharge in many cases, which enhances patient satisfaction and lowers healthcare costs [24,25,26]. In the present series, most patients (64.6%) underwent surgery under regional anesthesia with a mean operative time of 24.1 ± 7.1 min and minimal blood loss (23.8 ± 6.5 mL). No significant intraoperative or early postoperative complications were observed and most patients (98.8%) were discharged on the day of surgery. No deep pelvic dissection is needed with this unique device, so we cannot diagnose pelvic adhesions. However, no intraoperative anatomical vaginal distortion was observed. The POP-Q measurements, specifically point C for apical prolapse, point Ba for cystocele, and point Bp for rectocele, showed significant improvement six months postoperatively. A long-term follow-up was conducted through a telephone survey with a mean follow-up of 31.6 ± 8.3 months (range: 17 to 59 months). Out of 82 surveyed patients, 11 (13.4%) reported mild POP symptoms, with an average severity score of 3.9 on a 0–10 scale. Similarly, patient satisfaction rates were significantly high, with a mean score of 90.8 ± 17.1. Moreover, only two patients necessitated medical intervention for recurrent apical POP. One patient underwent a repeat surgical procedure, while the other was managed with a vaginal pessary.

The strengths of the present study include the presentation of one of the largest cohorts of patients undergoing repeated surgery for recurrent apical POP. Additionally, this is, to our knowledge, the first study to report long-term outcome results of a transvaginal approach for the surgical management of recurrent apical POP. All patients underwent a transvaginal minimally invasive SSL fixation using the EnPlace^®^ device. This surgical technique allows for the restoration of apical support under regional anesthesia and does not involve the use of synthetic mesh or extensive dissection. However, the study does have some limitations. The patient population exhibited a relatively homogenous character, and all participants were treated by highly experienced surgeons. This lack of diversity raises concerns about the generalizability of the study results to a broader patient demographic or to cases managed by less experienced surgeons.

## 5. Conclusions

Minimally invasive SSL fixation using the EnPlace^®^ device demonstrates favorable safety and efficacy for recurrent apical POP, offering a viable alternative to more invasive procedures with high patient satisfaction and low recurrence rates. There is a significant lack of clinical data and long-term follow-up regarding the efficacy and safety of surgical techniques for the repair of recurrent apical POP. In determining the most suitable surgical approach, surgeons must carefully consider anatomical distortions, complications related to prior mesh use, and individual patient factors. The ongoing debate about mesh use, surgical techniques, and patient-centered care principles emphasizes the need for further research to determine the best management strategies for recurrent apical POP.

## Figures and Tables

**Table 1:** Demographic and clinical characteristics.

Mean ± SD, or N (%)	Total N = 82	Previous Hysterectomy N = 57 (69.5%)	Previous Uterine Preservation N = 25 (30.5%)
Age (years)	65.9 ± 8.6	66.1 ± 8.2	65.6 ± 9.7
Parity	3.3 ± 1.8	3.3 ± 1.9	3.2 ± 1.3
BMI (kg/m^2^)	27.2 ± 3.7	28.1 ± 2.2	26.8 ± 1.9
Comorbidities	32 (39%)	23 (28%)	9 (36%)
Smoker	4 (4.8%)	1 (1.8%)	3 (12%)
Previous apical repair:			
Vaginal Hysterectomy	57 (69.5%)		
Vaginal mesh	19 (23.2%)		
SSL fixation	4 (4.9%)		
Manchester	2 (2.4%)		
Interval between primary surgery and recurrent repair (years)	3.2 ± 2.6	3.5 ± 3.1 *	2.1 ± 1.6 *
Dyspareunia	1 (1.2%)	0	1 (4%)
SUI	16 (19.5%)	11 (19.3%)	5 (20%)
OAB	22 (26.8%)	15 (26.3%)	7 (28%)
Defecation disorder	2 (2.4%)	1 (1.8%)	1 (4%)

BMI—body mass index, SSL—sacrospinous ligament, POP—pelvic organ prolapse, SUI—stress urinary incontinence, OAB—overactive bladder. * *p* = 0.039.

**Table 2:** Preoperative and postoperative POP-quantification (POP-Q) measurements.

	Point C (cm)	Point Ba (cm)	Point Bp (cm)
**Pre-operative**			
Total (N = 82)	+2.2 ± 0.9	+1.4 ± 2.1	+0.7 ± 1.4
Previous hysterectomy	+2.3 ± 1.6	+1.1 ± 2.4	+0.9 ± 1.5
Previous uterine preservation	+2.1 ± 0.6	+1.4 ± 1.3	+0.4 ± 1.0
**Six-week Follow-up**			
Total (N = 82)	−5.5 ± 0.8	−2.7 ± 0.5	−2.9 ± 0.3
Previous hysterectomy	−5.5 ± 0.6	−2.7 ± 0.5	−2.9 ± 0.3
Previous uterine preservation	−5.5 ± 1.3	−2.7 ± 0.4	−2.9 ± 0.2
**Six-month Follow-up**			
Total (N = 82)	−5.1 ± 1.3	−2.4 ± 0.9	−2.8 ± 0.6
Previous hysterectomy	−5.2 ± 1.1	−2.3 ± 0.8	−2.7 ± 0.5
Previous uterine preservation	−5.1 ± 0.3	−2.6 ± 0.9	−2.8 ± 0.7

**Table 3:** Postoperative subjective satisfaction scores at follow-up.

Satisfaction Score Mean ± SD, or N (%)	6 Weeks Follow-Up	6 Months Follow-Up	Long-Term Follow-Up
Total (0–100)	95.4 ± 8.4	93.5 ± 11.0	90.8 ± 17.1
≥90	74 (90.2%)	69 (84.2%)	69 (84.2%)
61–89	7 (8.6%)	10 (12.2%)	6 (7.3%)
≤60	1 (1.2%)	3 (3.6%)	7 (8.5%)

## Data Availability

Data is unavailable due to privacy or ethical restrictions.
